# Prospective verification of sonographic fetal weight estimators among term parturients in Uganda

**DOI:** 10.1186/s12884-021-03645-4

**Published:** 2021-03-04

**Authors:** Senai Goitom Sereke, Richard Okello Omara, Felix Bongomin, Sarah Nakubulwa, Harriet Nalubega Kisembo

**Affiliations:** 1 Department of Radiology and Radiotherapy, School of Medicine, Makerere University College of Health Sciences, Kampala, Uganda; 2 Department of Medicine, School of Medicine, Makerere University College of Health Sciences, Kampala, Uganda; 3 Department of Obstetrics and Gynecology, School of Medicine, Makerere University College of Health Sciences, Kampala, Uganda; 4 Department of Radiology, Mulago National Referral Hospital, Kampala, Uganda

**Keywords:** Fetal weight, Estimation, Formulas, Uganda

## Abstract

**Background:**

Accuracy of fetal weight estimation by ultrasound is essential in making decisions on the time and mode of delivery. There are many proposed formulas for fetal weight estimation such as Hadlock 1, Hadlock 2, Hadlock 3, Hadlock 4 and Shepard. What best applies to the Ugandan population is not known since no verification of any of the formulas has been done before. The primary aim of this study was to determine the accuracy of sonographic estimation of fetal weight using five most commonly used formulas, and analyze formula variations for different weight ranges.

**Methods:**

This was a hospital based prospective cohort study at Mulago National Referral Hospital, Kampala, Uganda. A total of 356 pregnant women who consented and were within 3 days of birth were enrolled. Prenatal ultrasound fetal weight determined by measuring the biparietal diameter, head circumference, abdominal circumference, femoral length, and then was compared with actual birth weight.

**Results:**

The overall accuracy of Hadlock 1, Hadlock 2, Hadlock 3, Hadlock 4 and Shepard formula were 66.9, 73.3, 77.3, 78.4 and 69.7% respectively. All Hadlocks showed significant mean difference between weight estimates and actual birth weight (*p* < 0.01) whereas Shepard formula did not [p - 0.2], when no stratification of fetal weights was done. However, all Hadlocks showed a none significant (*p*-values > 0.05) mean difference between weight estimates and actual birth weight when the actual birth weight was ≥4000.0 g. Shepard weight estimates showed a none significant mean difference when actual birth weight was < 4000 g. Bland-Altman graphs also showed a better agreement of weight estimated by Shepard formula and actual birth weights.

**Conclusion:**

All the five formulas were accurate at estimating actual birth weights within 10% accuracy. However, this accuracy varied with the fetal birth weight. Shepard was more accurate in estimating actual birth weights < 4000 g whereas all Hadlocks were more accurate when the actual birthweight was ≥4000 g.

**Supplementary Information:**

The online version contains supplementary material available at 10.1186/s12884-021-03645-4.

## Background

Maternal and infant mortality is a major public health issue globally and in Uganda. In 2017, an estimated 295,000 women died during and following pregnancy and child birth, as a vast majority of these deaths (94%) occurred in low resource setting. Sub-Saharan Africa alone accounted for approximately two-third (196,000) of maternal deaths and 6.000 of these cases were from Uganda [1].

Antenatal care reduces both maternal and infant morbidity and mortality and prenatal fetal weight estimation is known to be an important component of standard antenatal care. Fetal weight is one of the determinants of outcome of pregnancies and is also the major determinant of infants’ wellbeing in the first year of life [2]. Maternal risks associated with the delivery of an excessively big fetus include birth canal and pelvic floor injuries, as well as postpartum hemorrhage [3].

Accurate prediction of fetal weight is useful in the management of labor and reduces complications associated with macrosomia, thereby reduces maternal and peri-natal morbidity and mortality [4]. Ultrasound is widely used preferentially over the clinical models of fetal weight estimation prenatally and excludes fetal anomalies among preterm fetuses [5]. Although sonographic fetal weight could be predicted on single fetal parameter such as, the Biparietal Diameter (BPD), Abdominal Circumference (AC), Femur Length (FL), Head Circumference (HC) and Gestational age, a combination of several fetal parameters yields more accurate estimates of fetal weights [6, 7].

Fetal weight is influenced by several factors including maternal characteristics (age and weight), fetus related (anteriorly located placentae, oligohydramnios) and racial factors. Racial variation is an important factor to consider when sonographic fetal weight prediction models derived from one ethnic population are being used. All ultrasound scanners in Africa are imported from countries like America, Europe and Asia whose weight models are based on their population [8, 9]. Most fetal weight formulae are based on the normal fetal growth curves and do not take into consideration the factors attributing to either a growth restricted or macrosomic (> 4000 g) fetus. Thus, the accuracy of fetal weight estimations, in predicting hypothrophic and macrosomic fetuses using fetal growth curves has been questioned [10, 11].

Obstetric sonographic assessment for the purpose of obtaining fetal biometric measurement to predict fetal weight has been integrated into the main stream of obstetric practice during the past four decades [12]. Using sonography to estimate fetal weight has limitation including but not limited to the following: maternal obesity, oligohydramnios, anterior placentation, reduced visualization of fetal body structure and inadequate trained personnel [12, 13].

In one large cohort study in Israel, to assess and compare the accuracy of twenty-three models of sonographic fetal weight estimation, they found greater accuracy for models that used 3 or more fetal biometrics. These biometric are FL, AC, BPD, and HC [14]. In contrary to this, another large cohort study, twenty-six models of sonographic fetal weight estimation were used and showed that fetal weight estimations based on AC alone were more accurate than models based on FL, AC + FL, or AC + BPD [15].

Almost all the currently used formulas for estimating fetal weight have significant degree of disparity, and various studies have been done to compare the accuracy of different methods of estimation. However, significantly reducing potential perinatal complications associated with labor of both small and excessively large fetuses requires, accurate estimation of fetal weight occurs before deliveries [12].

Mulago national referral and teaching hospital, Kampala, Uganda, gives emergency caesarean sections service to 2209 mothers every 4 months of which 24.2% due to obstructed labor, 3.6% big baby, 5.8% cephalopelvic disproportion. (unreported Mulago national referral and teaching hospital labor suite records of 2018). Obstetricians seem to depend highly on fetal weight estimation by ultrasound to make decision in regard to the mode of delivery. To date, in Uganda, there is no formula that has been recommended for use across entire birth weight ranges due to lack of verification information. Moreover, data on the weight of Ugandan fetuses are scarce.

This study was, therefore, carried out to sonographically estimate fetal weights, in a population of Ugandan pregnant women, in a tertiary hospital in Kampala. Accuracy of fetal weight estimation using five most commonly used formulas (Hadlock 1, Hadlock 2, Hadlock 3, Hadlock 4, and SHEPARD) in the three strata of birth weights was determined and comparison of all the five formulas for weight estimation differences was done.

## Methods

### Study design and setting

This was a hospital based prospective cohort study conducted at the labor ward of Mulago National Referral and Teaching Hospital, Kampala, Uganda between January and June 2019. Mulago National Referral Hospital (Maternity Unit) is located in Kawempe 5 km from Kampala city center. The labor suite is managed by consultants, specialists, residents, intern doctors and midwives. There are minimum of seven midwives and eight doctors working in three and two shifts respectively daily. The unit admits on average 65 mothers for labor and conducts on average 17 Caesarian sections daily (Mulago labor suite register 2018).

### Study population

All consenting parturient mothers with singleton fetus at term pregnancy (37–42 weeks) and who delivered within 72 h from the onset of true labor were consecutively recruited. Deliveries resulting into still births, intrauterine fetal death, detectable congenital anomalies, severe oligo- or polyhydramnios and maternal diabetes or pre-eclampsia were excluded.

### Sample size

The sample size was determined using the Computer Programme for Epidemiologist (PEPI), version 3.01, described by Armitage and Berry, and 365 participants were determined as the sample size of the study. The following assumptions were used in determining the sample size, P = Taking the accuracy of Hadlock 4 formula for estimation of fetal weight to be on average 68%, Hadlock 3 formula 63.3%, Hadlock 2 formula 60.9% and Hadlock 1 formula 67.3%. Z = Z value (e.g. 1.96 for 95% confidence level). c = confidence interval, expressed as decimal/the margin of sampling error tolerated = 5%.

### Study procedure

**Fig. 1:**
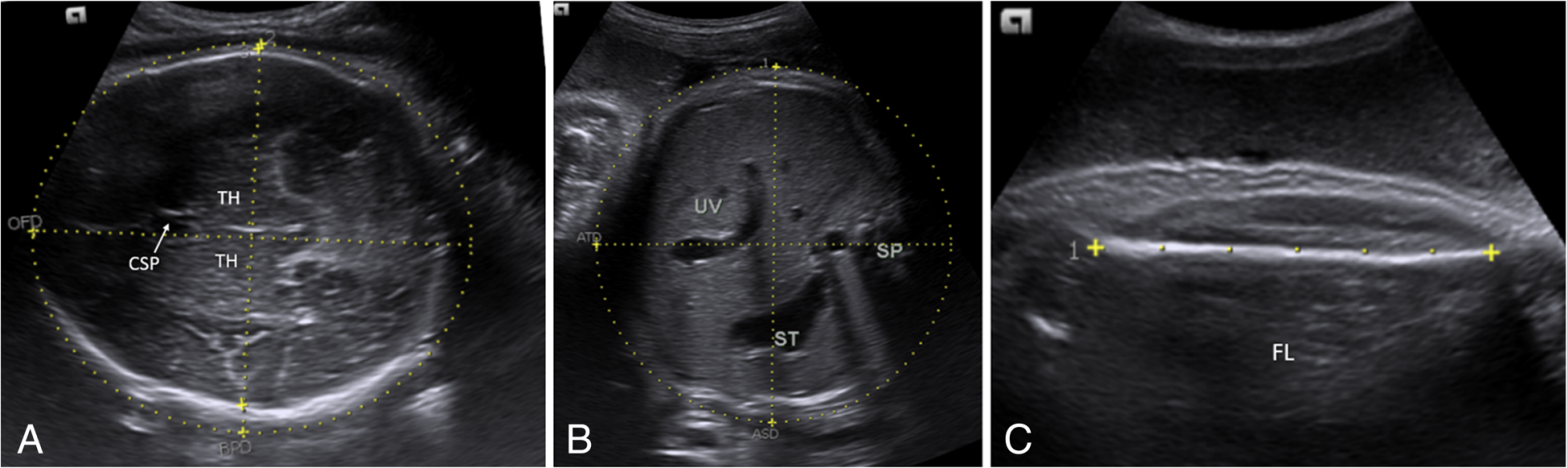
Sonographic parameters used to estimate fetal weight. B demonstrating the fetal head at the level of bithalamic (TH-thalamus), cavum septum pellucidum (CSP) and third ventricle. At this level was the BPD and HC taken **b** Abdominal circumference taken at the level of umbilical vein (UV), stomach (ST), spine (SP) and the posterior rib. **c** demonstrating femoral length measurement

Maternal data were gathered using pre-tested semi-structured questionnaire after the labor ward obstetrician confirmed active phase of labor and the parturient consented. The questionnaire used was developed for this study (Annex). Ultrasound examination of the fetus was performed by the principal investigator under the supervision of a qualified radiologist. A targeted ultrasound scan for biometry was performed using 3.5–5 MHz transducer on the SIEMENS ultrasound machine (ACUSON X600), model 10,789,636, manufactured June, 2016. Estimation of fetal weights was determined using measurements of fetal BPD, HC, AC and FL using standard recommended protocols [16, 17] (Fig. 1). AC and FL were used to calculate Hadlock 1; Hadlock 2 was calculated using BPD, AC and FL, Hadlock 3 using HC, AC and FL; Hadlock 4 BPD, HC, AC and FL; and Shepard BPD and AC. The estimated fetal weights in all the five formulas as well as the mean of each fetal parameter were stored in the ultrasound machine’s computer memory and immediately recorded in the datasheet.

After delivery, weights were taken using a standard analogue Way master (England) scale corrected for zero error within 30 min of delivery. The weights were rounded to the nearest 50 g.

### Statistical analysis

Normality test was applied first by visually comparing the respective histograms to a normal bell shaped graphs and then statistically tested for normality using Shapiro-Wilk Test. Absolute value of the difference between the sonographic estimated fetal weight by Hadlock 1, Hadlock 2, Hadlock 3, Hadlock 4, and Shepard formulas and the actual birth weight were calculated for each case and from this the mean weight difference and the percentage error were calculated. Mean percentage (relative) error was defined as: Estimated fetal weight (EFW) - actual birth weight (ABW)) ×  100/ABW, and absolute percentage error as (absolute value (EFW - ABW)) × 100/ABW. Percentage errors were grouped as being within 10, 20, 30% or more than 30% of the birth weight. Percentage error within 10% of the birth weight was considered accurate. The mean percentage error represented the sum of the positive (overestimation) and negative (underestimation) estimation from actual birth weight, and the mean absolute percentage error was the sum of the absolute deviation (regardless of their direction) reflecting the size of the overall predictive error in terms of actual birth weight. To compare sonographic fetal weight estimators and the actual birth weight, paired t-test was used. For significance in mean difference between sonographic weight estimators and the actual birth weight, a *p*-value of < 0.05 was considered. Comparison was also made at different categories of actual birth weight i.e. < 2500 g, 2500–3999 g, and ≥ 4000 g and placenta location.

Altman and Bland (B&A) analysis, was used to study the mean difference and construct limits of agreement. The B&A plot analysis was used to evaluate a bias between the mean differences, and to estimate an agreement interval, within which 95% of the differences of the Hadlock1, Hadlock 2, Hadlock 3, Hadlock 4 and Shepard formulas compared to actual weight points.

## Results

A total of 365 parturients were recruited in the study, 3 fetuses had congenital anomalies and 6 of the fetuses had a BPD ≥10 cm, and Shepard failed to calculate the fetal weight estimation and consequently was removed from this study, remaining 356 leading to a completion rate of 99.2%. The median age of the parturients was 24 years (IQR 21–29). The majority of the parturients were multiparous (49%). The maternal body mass index (BMI) in majority of the parturients were within the normal range (57.3%). The mean actual birth weight was 3.3 ± 0.42 kg (Range 1.6–4.35 kg). The median gestational age was 38 weeks 5 days (Range 38–42 weeks). Majority (91%) of the newborns were in the normal birth weight (2500–3999 g) (Table 1).

**Table 1:** Socio demographic characteristics of parturients in sonographic estimation of actual birth weight at Mulago national referral hospital

Median (IQR)	Variable
**Maternal age**	24(21–29)
**Gestational age**	38W5d (38–42)
**Gravidity**	**N (%)**
Primi	143 (40.2)
Multipara	176 (49.4)
Grand multipara	37 (10.4)
**Maternal BMI before labor**	
Under weight	2 (0.6)
Normal weight	204 (57.3)
Over weight	123 (34.6)
Obese	27 (7.6)
**Placentation**	
Anterior	147 (41.3)
Posterior	74 (20.8)
Fundal	134 (37.6)
**Categories of birth weight**	
Hypothrophic	21 (5.9)
Normal birth weight	324 (91.0)
Macrosomia	11 (3.1)

The maximum birth weight (4789 g) estimation was done by Shepard formula and the minimum birth weight (1634 g) estimated was by Hadlock 2. Hadlock 1 had the highest percentage (73%) of overestimation and Shepard had the highest percentage (52.8%) of underestimation of fetal birth weights (Table 2).

**Table 2:** Characteristics of estimators of birth weights of 356 neonates in Mulago national referral hospital

Formula	min	max	Under estim (n/%)	Over estimation (n/%)	**Mean ± SD
Hadlock 1	1642	4697			3358.9 ± 421.2
Hadlock 2	1634	4650			3290.0 ± 417.9
Hadlock 3	1714	4644			3243.2 ± 405.4
Hadlock4	1675	4531			3242.3 ± 404.6
Shepard	1750	4789			3161.4 ± 464.6
Hadlock1 diff	-861 g (under est)	944 g (over est)	96 (27.0)	260 (73.0)	175.3 ± 285.4
Hadlock2 diff	-915 g (under est)	960 g (over est)	122 (34.3)	234 (65.7)	106.4 ± 276.5
Hadlock3 diff	-910 g (under est)	977 g (over est	153 (43.0)	203 (57.0)	59.6 ± 281.0
Hadlock4 diff	− 922 g (under est)	976 g (over est)	142 (42.7)	204 (57.3)	58 ± 274.2
Shepard diff	− 1078 g (under est)	1028 g (over est)	188 (52.8)	168 (47.2)	−22.3 ± 327.0

**mean other than median was chosen as summary statistics because of the normality of sonographic estimators (formulas)

All the five formulas were able to estimate the actual birth weight with an accuracy of the difference being within 10%. The number of neonates with the difference between their actual birth weight and the formula lying within 10% accuracy for; Hadlock 1 was 238 (66.9%), Hadlock 2 was 261 (73.3%), Hadlock 3 was 275 (77.3%), Hadlock 4 was 279 (78.4%) and Shepard was 248 (69.7%). The mean difference between formulas and the actual birth weight was statistically significant for all the Hadlock formulas but for Shepard formula the difference wasn’t statistically significant (*p* = 0.200) (Tables 3 and 4).

**Table 3:** Accuracy of sonographic estimators in estimating actual birth weight of 356 neonates at Mulago national referral Hospital

Formula/percentage	(n / %)	MPE^a^ (mean ± SD)	APE (mean ± SD)
**HADLOCK 1**		6.0 ± 9.4	8.6 ± 7.0
within 10%	238 (66.9)		
within 10–20%	90 (25.3)		
within 20–30%	90 (25.3)		
≥ 30%	3 (0.8)		
**HADLOCK 2**		3.8 ± 8.9	7.4 ± 6.2
within 10%	261 (73.3)		
within 10–20%	78 (21.9)		
within 20–30%	14 (3.9)		
≥ 30%	3 (0.8)		
**HADLOCK 3**		2.3 ± 9.1	7.2 ± 5.9
within 10%	275 (77.3)		
within 10–20%	63 (17.7)		
within 20–30%	17 (4.8)		
≥ 30%	1 (0.3)		
**HADLOCK 4**		2.3 ± 8.9	7.0 ± 5.8
within 10%	279 (78.4)		
within 10–20%	63 (17.7)		
within 20–30%	13 (3.7)		
≥ 30%	1 (0.3)		
**SHEPARD**		−0.4 ± 10.3	8.1 ± 6.4
within 10%	248 (69.7)		
within 10–20%	88 (24.7)		
within 20–30%	16 (4.5)		
≥ 30%	4 (1.1)		

^a^MPE = predicted weight-actual weight/actual weight × 100

**Table 4:** Comparison of sonographic estimators with actual birth weight of 356 neonates in Mulago national referral hospital

Formula	mean diff	SD	t	95%CI	***p***-value	effect size/magnitude of mean diff
HADLOCK1^a^	175.3	285.4	11.6	(145.6–205.6)	< 0.001	0.61
HADLOCK2^a^	106.4	276.5	7.3	(77.6–135.2)	< 0.001	0.38
HADLOCK3^a^	59.6	281.0	4.0	(30.4–88.9)	0.0001	0.21
HADLOCK4^a^	58.7	274.2	4.0	(30.1–87.3)	< 0.001	0.21
SHEPARD^a^	22.3	327.0	1.3	(−11.8 to 56.3)	**0.200**	0.07

^a^paired t-test was used because the sonographic estimators (formulas) and actual birth weight were normally distributed, variance assumption was met and before and after weight measurements were taken

The mean percentage errors of the hypothrophic babies (< 2500 g) and normal birth weight babies (2500-3999 g) showed a steady decrement from Hadlock 1 to Hadlock 2 to Hadlock 3 to Hadlock 4 and to Shepard. And the mean percentage errors for macrosomic babies (≥4000 g) showed the reverse (Table 5 and 6). This implied, the Shepard formula followed by Hadlock 4 and Hadlock 3 are better estimators when the birth weight was in the range of hypothrophic to normal birth weight. Whereas Hadlock 1 followed by Hadlock 2 are better estimators if the estimated birth weights are in the macrosomic range. However, this couldn’t be conclusive because the different strata of birth weights were not fairly distributed (Table 6).

**Table 5:** Comparison of the mean percentage errors of the different formulas at different strata of birth weights

Estimator	MPE^a^, ABW [< 2500 g, ***n*** = 21]	MPE^a^, [ABW (2500-3999 g), ***n*** = 324]	MPE^a^, [ABW (≥4000 g), ***n*** = 11]
HADLOCK 1	11.4 ± 11.8	5.9 ± 9.1	−1.2 ± 6.4
HADLOCK 2	7.3 ± 10.5	3.8 ± 8.8	−2.9 ± 5.0
HADLOCK3	7.8 ± 11.5	2.2 ± 8.8	−4.3 ± 7.5
HADLOCK4	6.7 ± 10.7	2.2 ± 8.6	−4.3 ± 6.0
SHEPARD	−0.2 ± 10.6	−0.3 ± 10.4	−5.0 ± 7.3

^a^MPE = predicted weight-actual weight/actual weight × 100

**Table 6:** Comparison of Mean percentage errors (MPEs) of all the formulas at different strata of birth weight with MPE taken from other studies

Formula/ Strata of birth weights	Sereke et al. (this study) MPE	Australian study^a^ 2012 MPE	Southwest Nigeria study 2007 MPE
Shepard			
< 2500 g	−0.2 ± 10.6		
2500-3999 g	− 0.3 ± 10.4	−1.5 ± 9.1	
≥ 4000 g	−5.0 ± 7.3	−1.1 ± 10.1	
Hadlock 4			
< 2500 g	6.7 ± 10.7		
2500-3999 g	2.2 ± 8.6	−0.2 ± 7.5	
≥ 4000 g	−4.3 ± 6.0	3.7 ± 9.7	
Hadlock 3			
< 2500 g	7.8 ± 11.5		
2500-3999 g	2.2 ± 8.8	−1.7 ± 7.2	
≥ 4000 g	−4.3 ± 7.5	−2.6 ± 9.6	
Hadlock 2			
< 2500 g	7.3 ± 10.5		8.9 ± 3.1
2500-3999 g	3.8 ± 8.8	−1 ± 7.8	−2.2 ± 10.1
≥ 4000 g	−2.9 ± 5.0	−4.3 ± 10.2	−4.3 ± 6.9
Hadlock 1			
< 2500 g	11.4 ± 11.8		
2500-3999 g	5.9 ± 9.1	−1.3 ± 7.4	
≥ 4000 g	−1.2 ± 6.4	−2.9 ± 9.8	

^a^The Australia study had 102 babies in the normal birth weight category and 19 in the macrosomic category

The mean difference between all formulas (except SHEPARD) and actual birth weight was not statistically significant if the neonate weighed more than or equal to 4000 g. For neonates whose actual birth weight was less than 2500 g and between 2500 to 3999 g, the mean difference between the formulas and actually birth weight was statistically significant except for SHEPARD formula (Table 6).

**Fig. 2:**
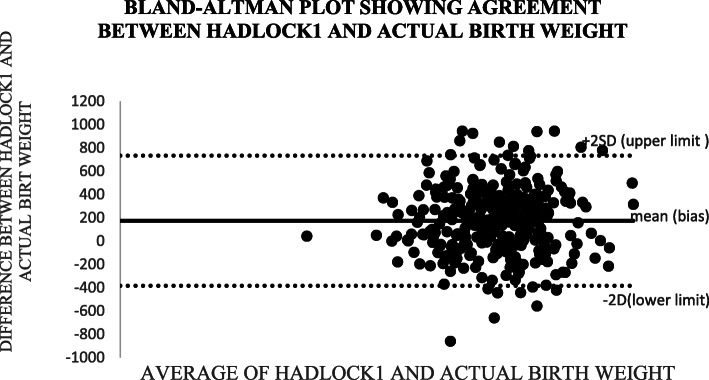
Plot showing agreements between HADLOCK1 fetal weight estimator and actual birth weight of 356 neonates at Mulago national referral hospital

**Fig. 3:**
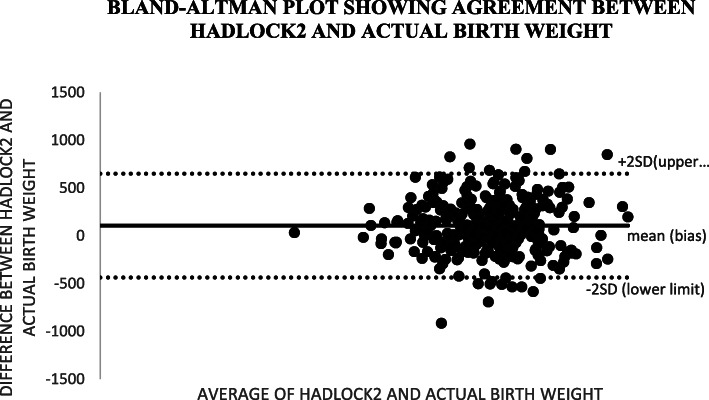
Plot showing agreements between HADLOCK2 fetal weight estimator and actual birth weight of 356 neonates at Mulago national referral hospital

**Fig. 4:**
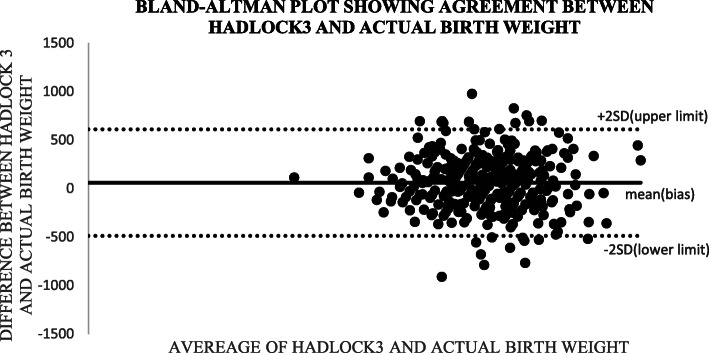
Plot showing agreements between HADLOCK3 fetal weight estimator and actual birth weight of 356 neonates at Mulago national referral hospital

**Fig. 5:**
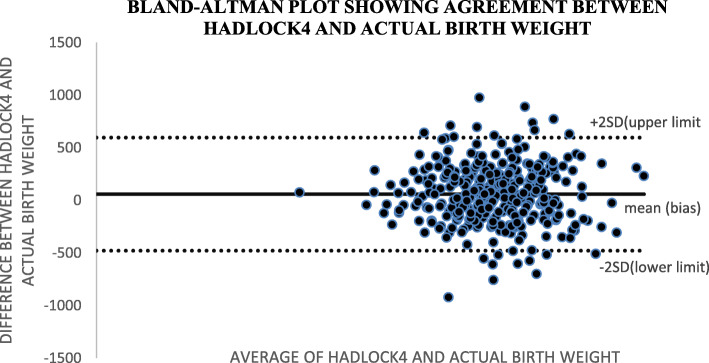
Plot showing agreements between HADLOCK4 fetal weight estimator and actual birth weight of 356 neonates at Mulago national referral hospital

**Fig. 6:**
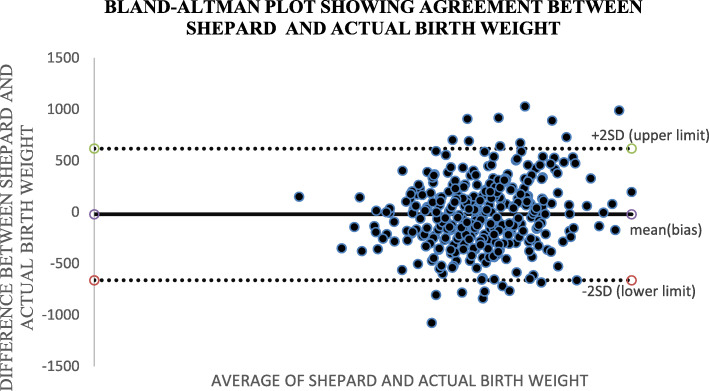
Plot showing agreements between SHEPARD fetal weight estimator and actual birth weight of 356 neonates at Mulago national referral hospital

In Bland and Altman plot, Hadlock 1, Hadlock 2, Hadlock 3 and Hadlock 4 were poor estimators of actual birth weight as shown in the plots. However, Shepard was a good estimator of actual birth weight (Figs. 2, 3, 4, 5 and 6).

## Discussion

Prenatal evaluation of fetal weight has a paramount importance in decision making in the management of ongoing pregnancies and the timing and mode of delivery. This study aimed at determining the most accurate fetal weight estimator for our population. We found that the mean actual birth weight was 3.3 ± 0.42 kg, the most accurate estimator (formula) of fetal weight was Shepard followed by Hadlock 4.

The maternal body mass index distribution was more than half in normal and one third in overweight. One study showed that fetal weight gain had linear increment with increased dependency on maternal obesity [18].

The parity had equal distribution between primiparous and multiparous, but very few with grand multiparous mothers. Increased parity was associated with underestimation of fetal weights when Combs [19] and Sabbagha [20] formulas were used but showed improvement with Hadlocks [21].

The mean actual birth weight was 3.3 ± 0.42 kg. This was similar to the southeast Nigerian study (3.3 ± 0.55 kg) [22], southwest Nigerian study (3.25 ± 0.66 kg) [12], Jordanian study (3.13 ± 0.5 kg) [23], Israeli study (3.32 ± 0.5 kg) [21] but slightly lower than Australian study (3.6 ± 0.3 Kg) [10].

In our study we used the numbering of the Hadlocks which is most commonly used. However, other authors used different labelling (alphabets) of Hadlock formula as in the study done by Campbell Westerway [10].

All formulas differed significantly in terms of the mean systematic percent error (*P* < 0.05). However, all the formulas estimated fetal birth weight with high accuracy within 10% of actual birth weight. Hadlock 4 being the highest (78.5%) and Hadlock 1 (66.9%) being the least which is comparable with the study done by Oshri et al., except the difference in percentages. Hadlock 1 was the least accurate (57.8%) and Hadlock 4 was the highest accurate (72.3%), Hadlock 2 (63.3%) and Hadlock 3 (68%) lied between those percentages [14]. Shepard formula was not included in Oshri et al. study. Comparisons of accuracy with 10% of actual birth weight in different studies has been reported in the literature (Table 7).

**Table 7:** Comparison of the fetal weight estimation formula taken from different studies measuring estimates within ABW ±10%

Fetal parameters	Formula	Year	Sereke et al. (this study) (%)	Israel study 2012 (%)	Jordanian study2012 (%)	German study, 2014 (%)	Southwest Nigeria 2007 (%)	Southeast Nigeria study 2014 (%)
BPD/AC	Shepard	1982	69.7			65.02		
BPD/HC/AC/FL	Hadlock 4(C)	1984	78.4	72.3				
HC/AC/FL	Hadlock 3(B)	1983	77.3	65.7	69.9			
BPD/AC/FL	Hadlock 2(D)	1985	73.3	60.5		66.43	68	67.5
AC/FL	Hadlock 1(A)	1982	66.9	57.8		62.21		

As much as all the formula showed high accuracy of estimation of fetal weight within 10% of actual birth weight, Shepard formula showed the best estimation of fetal weights with highest percentage of agreement with actual birth weights and smallest magnitude of mean difference (0.07) and the least percentage of error, followed by Hadlock 4 and Hadlock 3. The least percentage of agreement was demonstrated by Hadlock 1 (0.61).

The mean difference between all formulas (except SHEPARD) and actual birth weight was not statistically significant if the neonate weighed greater or equal to 4000 g. For neonates whose actual birth weight was less than 2500 g and between 2500 to 3900 g, the mean difference between the formulas and actually birth weight was statistically different except for SHEPARD. This is in consonance with the southeastern Nigeria study demonstrated with Hadlock 2. As the weight increased from normal birth weight to macrosomia, the accuracy of Hadlock 2 in estimation fetal weight increased significantly [12, 22]. Similarly, in 282 women in tertiary hospital of Logos Nigeria [24], Hadlock 3 demonstrated incremental percentage of accuracy within 10% of ABW as the weight increased from normal birthweight to macrosomia. Same result was also obtained from southwestern Nigeria [12].

Different studies showed different values of MPEs and APEs of the different formulas. Our study in contrast to the comparative studies done showed varying results in the MPEs of all the formulas. In contrary to our findings, German study [25] demonstrated that the Shepard was having better estimation of fetal weight in the macrosomic babies than the Hadlock 1 and Hadlock 2, as demonstrated by the least mean percentage error (− 1.8). The Israeli study on the other hand showed similar findings to this study that Hadlock 4 followed by Hadlock 3 showed better estimation of fetal weight though Shepard was not evaluated in the study. The comparisons of mean percentage errors for the different formulas from different studies are done (Table 8).

**Table 8:** Comparison of Mean percentage errors (MPE) of all the formulas with MPE taken from other studies

Formula/Model	Sereke et al. (this study) MPE	Israel study 2012 MPE	Jordanian study 2012 MPE	German study 2014 MPE	Southwest Nigeria 2007 MPE	Australian study 2012 MPE	Southeast Nigeria MPE
Shepard	−0.4 ± 10.3			−1.88		−1.4 ± 9.4	
Hadlock 4	2.3 ± 8.9	3.9 ± 16.7		(Merz^a^) -3.97		2.5 ± 8.1	
Hadlock 3	2.3 ± 9.1	5.7 ± 16.5	6.2 ± 5.3	(Warsof^b^) -6.44		−1.9 ± 7.8	
Hadlock 2	3.8 ± 8.9	7.0 ± 16.7		−5.40	−1.4 ± 9.88	−2.5 ± 8.4	5.1 ± 12.51
Hadlock 1	6.0 ± 9.4	7.4 ± 17.2		−6.94		−2.1 ± 8.0	

^a^Merz is one of the models used in the German study. The result is for Merz, not for Hadlock 4. Hadlock 4 wasn’t evaluate in the study

^b^Warsof is one of the models used in the German study. The result is hence for Warsof, not for Hadlock 3

The mean percentage errors in different strata of birth weight compared with MPE done on Australia by Westerway showed that Shepard better estimated macrosomic babies when compared to this study. On the other hand, Hadlock 4 better estimated fetal weights in the normal birth weight category than the macrosomic babies. The MPEs of different strata (Category) of birth weights are compared with other studies.

### Study limitation

Our study is not without limitations. A major limitation of this study is the lack of generalizability of the study findings since the study was conducted in one study site and due to none probability sampling method (consecutive sampling) used. However, this lack of generalizability could be minimal because of the referral nature of the Hospital where the study was conducted. Furthermore, Shepard formula can’t estimate fetal weight if the BPD is ≥10 cm. This resulted in to lack of estimation of six fetuses and consequently exclusion from the study. This exclusion could have introduced selection bias however this was minimal since the number excluded was less than 10%. Information bias in this study was minimal because the weighing scale used to measure actual birth weight was calibrated.

### Strength of the study

However, the strength of our study lies in the fact that we compared five sonographic fetal weight estimation formulas to determine which algorithm may be more valid in the population. To the best of our knowledge, this is the first study to do so in Uganda. Moreover, most of the published studies in the literature heavily relied on retrospective design. Our study however is of a prospective design. It had a relatively bigger sample size compared with previously published prospective studies we could get from the literature.

## Conclusion

All five formulas showed good accuracy in determining fetal weight. However, Shepard formula was more accurate in estimating the small (hypothrophic) and normal birth weight categories while Hadlock 4 for babies with macrosomic birth weight category. Therefore, in Uganda, Shepard (BPD and AC) formula may be used for estimation of hypothrophic and normal birth weights and Haldlock 4 (BPD/HC/AC/FL) for macrosomic babies. However, future, large, multicenter studies to confirm our findings are welcome.

## Supplementary Information

**Additional file 1.**

## Data Availability

The datasets used and/or analyzed during the current study are available from the corresponding author on reasonable request.

## Abbreviations

ABW: Actual birth weight
APE: Absolute percentage error
AC: Abdominal Circumference
A&B: Altman and Bland
BMI: Body mass index
BPD: Biparietal Diameter
EFW: Estimated fetal weight
FL: Femur Length
HC: Head Circumference
IQR: Interquartile range
MPE: Mean percentage error
