# Supplementary material for: Prospective verification of sonographic fetal weight estimators among term parturients in Uganda
Source: BMC Pregnancy Childbirth. 2021 Mar 4;21:175. doi: 10.1186/s12884-021-03645-4 (PMC7934251; doi:10.1186/s12884-021-03645-4)
Supplement: Supplementary file 1 — Additional file 1. [file 12884_2021_3645_MOESM1_ESM.doc]

## **Questionnaire and data collection tool**

**TITLE: Validation of sonographic fetal weight estimators among term parturients in uganda**

In this questionnaire, the research assistants will either fill in or select from a list of all coded diagnoses, procedures and free text.

Thank you for accepting to participate in this study. The information given shall be treated with utmost confidentiality. I will not put your name on it and feel free to give your own answer in order to address some problems encountered in our society.

Research assistant’s name…………………..……………………………………

Participant’s number……………………………………………………..……….

Telephone number…………………………………………..…………………….

Date ……………………………………………………………………………...

Time ………………………………………………..…………………………….

**A: Social demographics**

1. Participant’s age in years………..……
2. Tribe……………………………..……

**B: Obstetric factors**

**Date of data collection:** _______________ **Time**: ­­­­­________

1. Gravidity………………………………...………
2. Gestational age by LMP (wks)………………………..
3. Height of mother (meters)………………………….…….
4. Weight of mother (Kgs)…………………………………
5. Maternal Gestational or chronic Diabetes □ Yes □ No
6. Pre-eclampsia □ Yes □ No
7. Fetal presentation □ cephalic □ breech
8. Placentation □ anterior □ posterior □ fundal □ previa
9. Any anatomic gross abnormality? □ Yes □ No
10. Biparietal diameter (BPD) in cm ……………..
11. Head Circumference (HC) in cm ……………..
12. Abdominal Circumference (AC) in cm ………
13. Femoral Length (FL) …………
14. HADLOCK 1 estimated weight in gm……………..…
15. HADLOCK 2 estimated weight in gm
16. HADLOCK 3 estimated weight in gm
17. HADLOCK 4 estimated weight in gm
18. SHEPARD estimated weight in gm
19. Gestational Age by ultrasonography in weeks

**Delivery Date**: ____________________ **Time:** ___________

1. Outcome □ Alive □ dead
2. Sex of neonate □ boy □ girl
3. Measured birth weight (g)……………….
4. Duration from fetal weight estimation to delivery?...............hrs
